# Role of Matrix Metalloproteinases in Angiogenesis and Cancer

**DOI:** 10.3389/fonc.2019.01370

**Published:** 2019-12-06

**Authors:** Saray Quintero-Fabián, Rodrigo Arreola, Enrique Becerril-Villanueva, Julio César Torres-Romero, Victor Arana-Argáez, Julio Lara-Riegos, Mario Alberto Ramírez-Camacho, María Elizbeth Alvarez-Sánchez

**Affiliations:** ^1^Multidisciplinary Research Laboratory, Military School of Graduate of Health, Mexico City, Mexico; ^2^Psychiatric Genetics Department, National Institute of Psychiatry “Ramón de la Fuente”, Clinical Research Branch, Mexico City, Mexico; ^3^Psychoimmunology Laboratory, National Institute of Psychiatry “Ramón de la Fuente”, Mexico City, Mexico; ^4^Biochemistry and Molecular Genetics Laboratory, Facultad de Química de la Universidad Autónoma de Yucatán, Merida, Mexico; ^5^Pharmacology Laboratory, Facultad de Química de la Universidad Autónoma de Yucatán, Mérida, Mexico; ^6^Centro de Información de Medicamentos, Facultad de Química de la Universidad Autónoma de Yucatán, Mérida, Mexico; ^7^Genomic Sciences Graduate Program, Universidad Autónoma de la Ciudad de Mexico, Mexico City, Mexico

**Keywords:** angiogenesis and cancer, immune system, metalloproteinases, MMP, MT-MMP

## Abstract

During angiogenesis, new vessels emerge from existing endothelial lined vessels to promote the degradation of the vascular basement membrane and remodel the extracellular matrix (ECM), followed by endothelial cell migration, and proliferation and the new generation of matrix components. Matrix metalloproteinases (MMPs) participate in the disruption, tumor neovascularization, and subsequent metastasis while tissue inhibitors of metalloproteinases (TIMPs) downregulate the activity of these MMPs. Then, the angiogenic response can be directly or indirectly mediated by MMPs through the modulation of the balance between pro- and anti-angiogenic factors. This review analyzes recent knowledge on MMPs and their participation in angiogenesis.

## Introduction

### Epithelial-Mesenchymal Transition (EMT) in Metastasis and Migration

Currently, cancer research is focused on understanding the functional mechanisms underlying cell transformation and tumor progression that can be used to develop new markers and therapies (1). Cancer metastasis, the final step of tumor progression and the leading cause of cancer morbidity and mortality, involves the spread of cancer cells from the primary tumor to nearby tissues and distant organs; it is mediated by complex molecular changes of in cell cycle regulation (2, 3). The molecular changes that regulate the cell morphology and functions of epithelial cells, that is epithelial-mesenchymal transition (EMT), include the destruction of intercellular relationships and cell-matrix adhesive characteristics, extracellular matrix (ECM) breakdown, and cleavage of basement membrane components by matrix metalloproteinase (MMP) activity modulation. For example, when epithelial cells lose their polarity through EMT, cell-cell tight junctions and adhesive connections are lost, resulting in infiltration and an enhanced migration ability of these cells (4, 5). Therefore, EMT enables malignant cells to become motile and invasive, which constitutes a fundamental requisite for cancer metastasis (6).

On the other hand, angiogenesis, in which MMP participation is well-recognized, was found to be involved in cancer metastasis over 45 years ago. Interest in angiogenesis related to cancer arose in 1968 when it was highlighted that tumors secrete a diffusible substance that stimulates angiogenesis (7). It is now recognized that angiogenesis plays a crucial role in the establishment of cancer and is the rate-determining step in tumor progression (7, 8). Numerous studies have demonstrated the key participation of MMPs along with EMT to promote angiogenesis, infiltration by cancer cells, and metastasis (9–13). MMPs are a family of zinc-binding metalloproteinases that participate in the degradation of ECM components, including the basement membrane and the tumor surface, resulting in tumor cell migration into the near tissue. Furthermore, MMPs promote tumor growth and spread through the capillary endothelium and neovascularization (14).

Given the relevance of MMPs in diseases such as cancer, this work presents the most representative studies on the subject. We emphasize the role of cytokines and growth factors inducing EMT in various types of cancer together with the role of MMPs. We also analyzed the carcinogenic and angiogenic processes, and with the participation of MMPs, cytokines, and immune system cells in these processes along with the regulation, activation, and signaling pathways of MMPs in cancer cells.

## Biochemical Properties of Matrix Metalloproteinases

**Figure 1:**
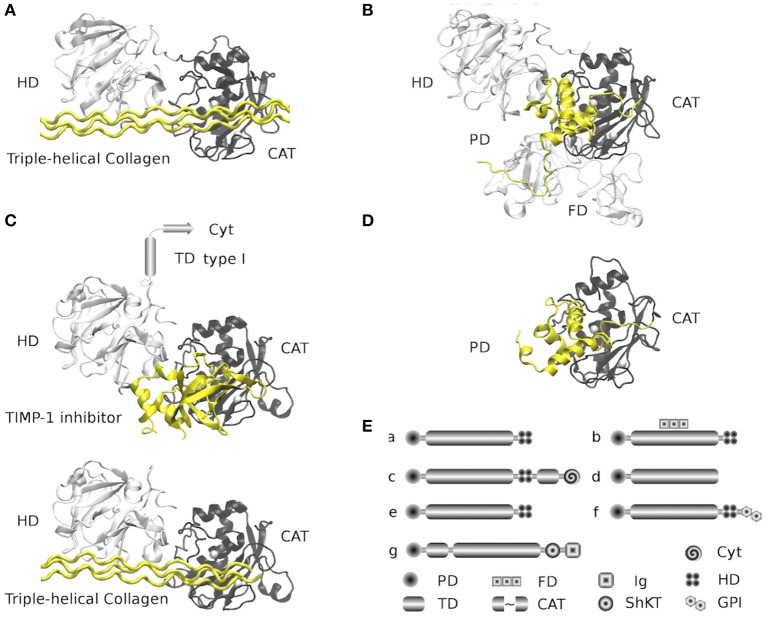
Structure and architectures of MMPs. The selected Protein Data Bank (PDBs) structures are comprehensive (when possible) full-length peptides found in the available coordinates files, all structures were overlapped at similar positions. For every structure, the propeptide domain and triple-helical collagen peptide appear in yellow, while the catalytic domains (right) appear in black, and hemopexin domains (left) in white. **(A)** MMP-1 family (collagenases and stromelysins) is represented by the structure of the MMP-1 from Human (PDB: 4AUO) in complex with triple-helical collagen peptide. Family members: MMP-1, MMP-8, MMP-13, MMP-3, MMP-10, MMP-12, MMP-20, and MMP-27. **(B)** Gelatinases family is represented by the full-length structure of the inactive MMP-2 with propeptide from Human (PDB: 1CK7). The additional fibronectin type II domains appear in white and are located under the catalytic domain (black). Family members: MMP-2 and MMP-9. **(C)** MT-MMPs transmembrane type I family. Represented by two structures mixed in two models of MMP-14 (MT1-MMP) from Human (PDBs: 2MQS and 3MA2). Models were built by the superposition of the homologous structure of MMP-1 (PDB: 4AUO). 2MQS structure is a complex of the hemopexin domains with triple-helical collagen peptide; 3MA2 structure is a complex of the catalytic domain with TIMP-1 inhibitor. The models show the hypothetical MMP-14 with hemopexin and catalytic domains in complex with TIMP-1 and triple-helical collagen peptide. The structure of helical membranal fragment is unknown (542–562) and the structure of the cytoplasmatic tail of the C-terminal fragment (563–582) is available in a complex with the FERN domain from Radixin (PDB: 3X23, structure not represented). Family members: MMP-14 (MT1-MMP), MMP-15 (MT2-MMP), MMP-16 (MT3-MMP), and MMP-24 (MT5-MMP). **(D)** Matrilysin family (shortest MMPs). Represented by the full-length structure of the inactive MMP-7 with propeptide from Human (PDB: 2MZE). This family lacks hemopexin domains. Family members: MMP-7 and MMP-26. **(E)** Global MMPs architecture by families. Families (a–d) are represented from **(A,D)**. (e) is the MMP stromelysins type 3 family (structures available but not complete); the architecture is similar to that of MMP-1 family. Family members: MMP-11 (stromelysin 3), MMP-21, MMP-28 and MMP-19 (evolutionary close to MMP-11 and MMP-7). (f) is the MT-MMP GPI (Glycosylphosphatidylinisotol) anchored family (structures not available), the architecture is similar to that of MMP-1 family and closely related to stromelysin type 3 family, but it is attached to the membrane by the GPI. Family members: MMP-17 (MT4-MMP), MMP-25 (MT6-MMP). The (g) family is represented by the MMP-23 (structures not available) and shares the catalytic domain with other families; the architecture is different on the N-terminal of the catalytic domain, containing a type II helical membrane fragment. On the C-terminal are an ShKT (Stichodactyla toxin) domain (with potential channel-modulatory activity) and an Ig-like (Immunoglobulin) C2-type domain that mediates protein-protein interactions. Cyt: cytoplasmatic domain, PD: Propeptide domain, TD: transmembrane helix, FD: Fibronectin type-II domains, CAT: zinc-dependent metalloproteinase domain, Ig: Ig-like C2-type domain and ShKT type domain. All figures were made with VMD (Visual Molecular Dynamics) (20).

**Figure 2:**
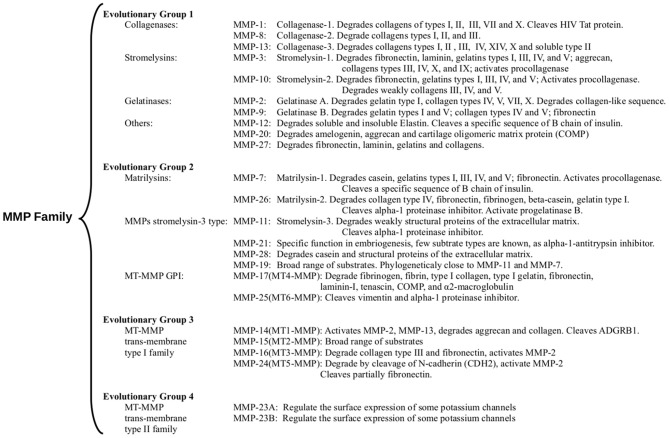
Evolutionary relationship of the catalytically domain of MMP family. Additionally, the main substrates are mentioned. MMPs classification is based on a phylogenetic tree of the catalytic domains reported (23). The sequences are arranged in four groups: (1) Evolutionary group 1 (Figures 1A,B) mainly assembles collagenases, stromelysins, and gelatinases, but other MMPs with a broad range of activities appear grouped. (2) Evolutionary group 2 (Figures 1D,Ee,f) mainly include matrilysins, the GPI-anchored MMPs, and other metalloproteinases as MMP-11 (a stromelysin) and MMP-21 (an MMP with a specific function in embryogenesis). (3) Evolutionary group 3 (Figure 1C) includes the MT-MMP trans-membrane type I family (MT1-MMP, MT2-MMP, MT3-MMP, and MT5-MMP). All three groups share a basic architecture with PD-CAT-HD domains array with a few additions or deletions, as matrilysins. The shortest MMPs without HD domain (group 3) contain a transmembrane type I helix and cytoplasmic domains after the HD domain (Figure 1). (4) We added evolutionary group 4 that includes the MT-MMP transmembrane type II family (Figure 1E) with MMP-23A and MMP-23B proteins. MMP-23A gene is considered a pseudogene produced by duplication of the MMP-23B gene. Sources: “GeneCards: the human gene database” (24) and Uniprot databases (25).

MMPs, also known as matrixins, are members of the metzincin protease superfamily of zinc-endopeptidases.They display a specific proteolytic activity against a broad range of substrates located on the ECM. Other members of the superfamily include A Disintegrin and metalloproteinases (ADAMs), and ADAMs with thrombospondin motifs (ADAMTSs), which contain a conserved methionine (Met or M) residue adjacent to the active site (15, 16). The first MMP (collagenase/MMP-1) was identified more than five decades ago (17). Since then, a total of 28 members have been named MMPs and given a distinctive numbering, and have been identified in vertebrates. In humans, there are 23 paralogs of MMPs (including a duplicated MMP-23 gene that encodes two identical forms of MMP-23), out of which at least 14 can be found expressed in the vascular endothelium (18, 19). The typical structure of MMPs consists of an N-terminal zymogenic propeptide domain (~80 amino acids), a metal-dependent catalytic domain (~170 amino acids), a linker region (~15–65 amino acids), and a C-terminal hemopexin-like domain (~200 amino acids) (Figure 1) (19, 21). MMP classification is traditionally centered on the substrate specificities observed and the common structural domain architecture. The MMP family can be divided into at least six subfamilies: (1) collagenases; (2) gelatinases, (3) stromelysins, (4) matrilysins, (5) MMP membrane-type (MT)-MMPs, and (6) other MMPs. However, since they present a wide range of substrates and different functions, many of these are similar but have a different biological function that has yet to be clarified. We used a classification related to their evolutionary origin to locate MMPs that have not been properly classified (21, 22) (Figure 2).

All MMPs are produced as proenzymes and require a proteolytic cleavage under physiological conditions to promote the release of the propeptide domain (zymogen activation) and generate mature MMPs (22). This means that the activity of MMPs is regulated by a post-translational proteolytic cleavage and endogenous inhibitors (15, 21). Nevertheless, efforts to define the substrate recognition profile by MMPs have resulted in substrate selectivity conferred by key subsite interactions (P3, P1′, P2′, and P3′) with a motif sequence specificity “P-X-X-|-L-X-X,” even though combined frequencies of subsites have been observed. It is known that subsite P3 maintains a high preference for Pro; still, many MMPs favor small residues (Ala/Val/Ile/Leu) and less frequent aliphatic residues. While subsite P1′ maintains hydrophobic residues with preferences for Leu/Ile/Val/Met, subsite P2′ maintains preference for Ile/Val, Glu/Asp, and Lys/Arg/His depending on the MMP. Finally, subsites P3′ and P2′ are inconsistent in all MMPs with any preference for Gly and Ala (26, 27). Therefore, the ability to recognize a wide variety of substrates selected by profile signatures by MMPs involves the peptide hydrolysis of latent protein targets, located on the ECM and the surface of the cell membrane.

Moreover, the MMP catalytic domain of the metzincin clan of metalloendopeptidases shares a general zinc-binding signature as core of the catalytic reactivity; the signature conserved sequence is the H-E-X-X-H-X-X-G-X-X-H/D region. Additionally, the conserved M residue of the superfamily is located on the methionine containing turn (Met-turn) which is part of the catalytic region and likely has structural-stability functions; nevertheless, the strict conservation of this residue remains unclear (28, 29) (Figure 1). All MMPs differ in expression, localization, substrate profile specificities, and structural organization. For further details about the structure and function of MMPs see (14, 15, 30).

## Cancer and Angiogenesis

Angiogenesis is a process by which new blood vessels or capillaries grow from the preexisting vasculature, and it is necessary for diffusion of nutrients and delivery of oxygen for tissue metabolism or cells involved in wound healing, myeloid and stromal cells. New blood vessels require the dismantling of endothelial lined vessels via the “sprouting” of endothelial cells (ECs), expanding the vascular tree (31). Moreover, the neo-vessel networks play more complex roles in diverse tissues such as the endometrium during the menstrual cycle, implantation, and endothelial cell migration out of the existing blood vessels (32). Given the complexity of a process as angiogenesis, the vascular endothelial growth factor, VEGF (VEGF-A), plays a remarkable role in signaling through the VEGF receptor-2 (FLK1) which induces angiogenesis in both health and disease processes. VEGF activity is enhanced by VEGF co-receptors, such as NRP1 and NRP2. In contrast, the loss of VEGF results in the interruption of vascular development. Placental growth factor (PlGF) is a cytokine VEGF homolog that stimulates angiogenesis and various types of cells, such as myeloids and stromals cancers, in addition to activating tumor cells, while their inhibition improves cancer treatment (33).

Collagenases (MMP-1, −8, and −13) are proteins associated with angiogenesis, and their loss leads to the irreversible rupture of the matrix. Type IV collagen participates in cell endothelial migration in the interstitial stromal spaces. It is known that the tissue inhibitors of metalloproteinases (TIMP-1, TIMP-2, TIMP-3, and TIMP-4) regulate them, playing a key role in angiogenesis regulation by inhibiting neovascularization (34).

In adults, angiogenesis is initiated only under inflammation or hypoxic conditions (35). In the early proliferative stage, vascular repair must predominate to control bleeding by vasoconstriction and coagulation. During menstruation, the endometrium is expelled if the ovule is not fertilized. Women who suffer from endometriosis show aggressive angiogenesis in the peritoneal cavity (36).

On the other hand, several studies have established the importance of transmembrane receptors and ligands participating in cell differentiation. Their role in endothelial sprouting during angiogenesis has recently been studied. ECs express several Notch receptors (such Notch1 and Notch4), as well as the Notch1 protein and Notch ligand delta-like 4 (DLL4), which are important signals for vascular development (37). In most of the healthy population, resting ECs showed long half-lives through VEGF activation, Notch signaling, and angiopoietin-1 (ANG-1) and fibroblast growth factors (FGFs) expression (33). Recent knowledge concerning the complexity of angiogenesis indubitably shows the role of the participants in this event and allows for finding applications in anti-angiogenic therapy.

As previously mentioned, angiogenesis is a normal development and part of the healing process; however, it is key to tumor branching and arborization under pathological conditions such as cancer. The formation of new vascular networks promotes the growth, maintenance, and spread of cancer (38). During angiogenesis in cancer, alterations have been described at the level of lymphangiogenesis and vasculogenesis, both processes are highly involved in the propagation of cancer cells and an unfavorable prognosis (39).

The accelerated growth of the tumor leads to hypoxic tumor microenvironment, interstitial hypertension, and acidosis. To reverse these adverse physicochemical changes, VEGF-C and VEGF-D are synthesized by the activation of VEGFR-3/2, triggering a rise in diameter and density of the peritumoral lymphatic vessels, favoring the propagation of tumor cells toward sentinel lymph nodes (40, 41). It has been shown that the inhibition of these factors by the use of antibodies decreases lymphogenesis and metastasis in nearby ganglia (42–44). Then, angiogenesis maintains a constant and permanent supply of nutrients for cancer cells that leads to tumor growth. This aberrant revascularization begins after the loss of regulation of inhibitory factors (e.g., thrombospondin-1) and angiogenic promoters (VEGF) (45, 46). Hypoxia-inducible factor (HIF) is one of the first growth factors to initiate the abnormal process of vascular growth and responds to the low oxygen tension in the tumor mass. Subsequently, a wave of growth factors such as EGF, basic and acidic FGF, estrogen, prostaglandin E1 and E2, IL-8, TGF, TNF, neuropilins, and VEGF promotes the formation of a vascular network that ensures the exchange of oxygen and nutrients with the tumor (5, 31, 47, 48). This vascularization process is regulated primarily by VEGF-A/VEGF-1,2 and DLL4 signaling. The activation of ECs also triggers a branching process toward the central region of the tumor (49, 50). This new supply and drainage network that supports the tumor allows the latter to maintain a favorable microenvironment for its growth and dissemination. At present, the tumor niche is considered an independent organ able to maintain itself (51). Additionally, integrin receptors are overexpressed in tumor ECs and play a key role connecting the cell cytoskeleton to the extracellular matrix protein ligands such as arginine-glycine-aspartic acid (RGD). This binding interaction between integrin and protein ligands is an important mechanism during the angiogenesis of tumor endothelial cells (52).

### MMPs in Cancer Angiogenesis

It is well known that MMPs have been implicated in angiogenesis regulation as well as in the anomalous relationship between cancer and the related processes of angiogenesis, vasculogenesis and lymphangiogenesis. MMPs also have a role in the immune system action in cancer development and progression (Tables 1, 2). The pro- and anti-angiogenic effects of MMPs participate in crucial steps as the ability to degrade ECM or cleave several substrates. Specifically, MMP-2 and MMP-9 give rise to the modulation of the dynamic remodeling of ECM (editing aggrecan, collagens, elastin, fibronectin, laminins, and glycosaminoglycans, and latent signaling proteins), activating and deactivating by proteolytic cleavages releasing biological activities that induce cellular regulation (108, 109). MMP activation can be induced by several angiogenic factors, such as VEGF, basic fibroblast growth factors (bFGF), TGF-α and -β, and angiogenin. Specifically, MMP-1 activity promotes the expression of the vascular endothelial growth factor receptor 2 (VEGFR2) and EC proliferation, stimulating serine/threonine-protein kinase MARK2 (PAR-1) and activating the transcription factor NF-κB, suggesting the existence of a mechanism by which MMP-1 stimulates vascular remodeling and angiogenesis (110). Similarly, MMP-7 modulates the VEGF pathway in human umbilical vein endothelial cells (HUVECs), degrading soluble VEGFR-1 and in turn promoting angiogenesis (111). TNF-α, IL-8 and other factors with a known pro-angiogenic capacity, stimulate the production of MMP-2,−8, and−9 in ECs and regulate the angiogenesis process (63, 112).

**Table 1:** Immune system proteins associated to MMPs in angiogenesis and cancer.

**Protein**	**Intervention/process**	**Tumor type/cell lines**	**MMP**	**References**
IL-1α, IL-3, VEGF, GCSF and GM-CSF	Secretion of proteins (IL-1α, IL-3, VEGF, GCSF, GM-CSF) under Hypoxia stress increase efficiency for induction of angiogenesis	Human A431 squamous carcino cells Human A549 non-small lung cells, and H1299 NSCL lung cells	Hypoxia increase secretion: MMP-13. Hypoxia increase secretion: MMP-3, MMP-9 and MMP-13	(53)
IL-1βTNF- α	IL-1β as inductor shows a slight dose-dependent increasing secretion of MMP-2. TNF-α as inductor shows a slight dose-dependent increasing secretion of MMP-9. A curious fact, retinoic acid strongly inhibited MMP-2 secretion	Human Glioblastoma T-98G cell line	MMP-2 and MMP-9	(54)
IL-1β	IL-1β induced MMP-2 and MMP-9 expression and activities mediated NK-kB activation, whereas melatonin suppresses it	Human Gastric adenocarcinoma MGC803cell line and Human Gastric cancer SGC-7901 cell line	MMP-2 and MMP-9	(19)
IL-1β	IL-1β/p38/AP-1(c-fos)/MMP-2 and MMP-9 pathway play an important role in metastasis in gastric cancer	Human Gastric cancer cell lines MKN45 and AGS	MMP-2 and MMP-9 increase gene expression and protein expression in response to IL-1β treatment	(55)
IL-1β	The STAT3 signaling is present in myeloid cells in human cancer angiogenesis and it is required for the cellular migration. The activity of STAT3 in tumor-associated myeloid cells participate in the elevated gene transcription of VEGF, bFGF, IL-1β MMP-9, CCL2 and CXL2	Murine Tumor-infiltrating myeloid cells	MMP-9 is elevate by the STAT3 activity	(23)
IL-5	L-5 increased migration and MMP-9 expression via activation of transcription factors NF-κB and AP-1, and induced activation of ERK1/2 and Jak-Stat signaling in both cells. IL-5Rα, inhibition, suppressed migration, ERK1/2, NF-κB, AP-1 activation and MMP-9 expression. MMP-2 expression remains without changes	Human Bladder carcinoma cell lines: 5637, T24 and HT1376	MMP-2 and MMP-9	(24, 25)
IL-22 (IL-10 family member) and IL-22R1	Promotes gastric cancer cell invasion through STAT3 and ERK signaling in MKN28 Promotes gastric cancer cell invasion activating AKT signaling in SGC-7901	Human Gastric cancer cell lines MKN28 and SGC-7901	IL-22 upregulate the gene expression of MMP-7 and MMP-13 in MKN28 IL-22 upregulate the gene expression of MMP-9 in SGC-7901	(56, 57)
IL-10	IL-10-stimulated macrophages polarized to M2 phenotype (low IL-12, IL-6 expression and IL-10 high expression) significantly increased AGS and RKO cells Invasion radio. Conditioned medium from IL-10-stimulated macrophages (M2) induced in AGS cell motility, migration and mediated angiogenesis	Human Diffuse gastric carcinoma cell line: AGS Colon carcinoma cell line: RKO	MMP-2 and MMP-9 elevated expression and activities on AGS cells with conditioned medium from IL-10-stimulated macrophages (M2)	(58)
IL-8 (CXCL8) IL-1β	Breast cancer cells secreting high levels of RANTES, CCL2 and G-CSF showing a potential capability to recruit monocytes and to instruct them to secrete high levels of IL-1β and IL-8, and MMP-1, MMP-2 and MMP-10	Patient samples diagnosed with ductal carcinoma. Monocytic Cell lines THP-1, U937, and Human Breast cancer cell lines: T47D (HTB-133), MCF-7 and MDA-MB-231	MMP-1, MMP-2 and MMP-10	(59)
IL-8 (CXCL8)	Co-cultured ovarian cancer stem-like cells with macrophages (derived from THP-1 cells) polarized to M2 phenotype increased IL-10, VEGF, MMP-9 and IL-8 secretion, and CD163 and STAT3 expression. THP-1 cell conditioned medium plus IL-8 induced stemness in SKOV3 cells involving IL-8/STAT3 signaling.	Human SKOV3-derived ovarian cancer stem-like cells.	MMP-9	(60)
IL-8 (CXCL8)	Recruited B cells mediated IL-8/androgen receptor and MMP signals in bladder cancer could enhance invasion and metastasis	Bladder tumor specimens were collected from 24 patients Human Bladder cancer cell lines TCCSUP, T24 and J82, and the Ramos B cell line	MMP-1 and MMP-9	(61)
IL-8 (CXCL8)	*Porphyromonas gingivalis* on chronic periodontitis promoted the invasive ability of carcinoma cells by up-regulation of IL-8, MMP-1 and MMP-2. Other MMPs are up-regulated too like MMP-7, MMP-9 and MMP-10	Human Oral squamous cell carcinoma cells SCC-25, OSC-20 and SAS cells	MMP-1, MMP-2, MMP-7, MMP-9 and MMP-10 are up-regulated after 72 hours of *P. gingivalis* infection	(62)
IL-8 (CXCL8)	IL-8 directly enhances endothelial cell survival, proliferation, MMP production and modulate angiogenesis	Human Umbilical Vein Endothelial Cell and dermal microvascular endothelial cells	MMP-2 and MMP-9 mRNA expression was increased in cells treated with 10 and 100 ng/ml IL-8. The Culture supernatant showed high level of both active MMPs	(63)
IL-8 (CXCL8) IL-6	IL-8, IL-9, MMP-2 and MMP-9 secreted by Falconi Anemia Cells are expressed under the control of NF-kB/TNF-α signaling pathways. These secretory factors are effective on promoting proliferation, migration, invasion of surrounding tumor cells	Falconi Anemia Cells (EUFA274, EUFA274Rev, EUFA450, EUFA450RevR, FANCD2 and FANCD2 corrected), MDA-MB-231 cells, PC3 cells, MCF7 cells, MCF10A cells	MMP-2 MMP-7 and MMP-9 are overexpressed	(64)
IL-8 (CXCL8) IL-1β, VEGF	Self-conditioned medium collected from A549 cells was treated with neutralizing antibodies against IL-1β, IL-8, and VEGF and used in A459 cells. The inhibition of motility and invasion in A549 cells were observed, the effect was higher in IL-8 and VEGF neutralizing medium	A549 (human lung adenocarcinoma), MCF-7 (breast adenocarcinoma) and HT-29 (colon carcinoma)	MMP-2 activity was detected in Self-conditioned medium collected from A549 cells	(65)
IL-8, VEGF, angiogenin, and NKG2D	Lung tumor–associated NK cells (TANKs) of peripheral blood and tumor-infiltrating NK cells (TINKs) induced functional angiogenesis-associated behaviors of endothelial cells *in vitro*. TANKs release TIMP-1, TIMP-2, and MMP-9, proteins involved in tissue remodeling and invasion process. They also increase the expression the angiogenin, VEGF and CXCL8, depending on tumor location	Human Lung tumor–associated NK cells (TANKs) of peripheral blood and tumor-infiltrating NK cells (TINKs) of patients with colorectal cancer	MMPs down-regulate the activator NKG2D, a surface marker for NK cell activation, in TANKs. Which is correlated with increased release of MMP-9, TIMP-1 and TIMP-2.	(66)
IL-8, IL-6, IL-1a, IL-1RA, GM-CSF, CCL5 (RANTES), TNF-α, VEGFA	Different lines cells from similar tumors show a varied secreted immunological biomarker profiles. Although almost every cells lines express the eight cytokines, apparently the metastatic stage, cellular origin, the site and the genome differences plus, an uncertain passage number of the cell lines, cause different profiles: SCC25 express mostly VEGFA and CCL5; SCC19 express mostly VEGFA, IL-6 and IL-8; SCC92 mostly express TNF-α IL-6, IL-1α and IL-8; SCC99 express mostly IL-8	Human Head and neck squamous cell carcinoma lines: SCC4, SCC15, SCC25, SCC84 and SCC92 are from the oral cavity; while SCC19 and SCC99 are from the oropharynx	MMP-1, MMP-7 and MMP-9 are higher expressed on SCC25. Others cell lines express different MMP profiles: SCC99 mostly express MMP-1 and MMP-9; SCC15, SCC19 and SCC84 mostly express MMP-7 and MMP-9; SCC4 and SCC92 mostly express MMP-9	(67)
IL-8	IL-8 and MMP-9 are co-expressed on MCF-7 cell line induced by TPA (a carcinogen). Orientin downregulates signal PKCα /ERK and blocks the nuclear translocation of AP-1 and STAT3 causing an attenuation of IL-8 and MMP-9 induced by TPA treatment, but only affected the migration and invasion of ER-positive MCF-7 cells	Human Breast cancer cell line MCF-7 estrogen receptor positive	MMP-9	(68)
IL-8, IL-6	MMP expression is regulated by cancer cell density via the signaling of IL-6 and IL-8. The synergistic signaling of IL-6 and IL-8 regulates the production of MMPs through the JAK/STAT signaling pathway	Human Fibrosarcoma HT1080 cells and breast carcinoma MDA-MB-231 cels	HT1080 in high cell density not only expresses MMP-1, MMP-2 and MMP-3 mainly but also MMP-11 and MMP-14 MDA-MB-231 in high cell density not only expresses MMP-14 mainly, but also MMP-7, MMP-1 and MMP-2	(69)
IL-6	In macrophages, the homeo-domain protein Six1 overexpression was able to induce IL-6 up-regulation and increase activity of STAT3 in Hepatocellular carcinoma cells. Macrophages Six1 upregulate IL-6 and MMP-9 and can stimulate cancer cell invasion by elevating MMP-9 expression	Human Leukemic monocyte cell line: THP-1; Human hepatoma cell line: A59T; and hepatocellular carcinoma cell line: HepG2 Two paraffin-embedded Hepatocellular carcinoma tissue arrays	MMP-9	(70)
IL-6	IL-6 regulates MMP expression via proximal GAS-like STAT binding elements (SBEs). IL-6 lead the formation of a complex STAT1/AP-1	Patient colon tumor tissue Human Colorectal carcinoma cell lines HT29, SW480, LISP-1, LIM1215, HCT116, and LS174T Hepatocyte-derived HepG2 cell line	MMP-1 and MMP-3	(71)
IL-6	IL-6/ NOS2 inflammatory signals regulate MMP-9 and MMP-2 dependent metastatic activity	Nasopharyngeal carcinoma from patients	MMP-9 and MMP-2	(72)
IL-6	IL-6 secreted by astrocytes induce upregulation of MMP-14 increasing migration and invasion of Glioma cell lines	Human Glioma cell lines U251 and A172 Astrocytes	MMP-14 (MT1-MMP)	(73)
IL-11	IL-11 promoting chronic gastric inflammation and associated tumorigenesis mediated by excessive activation of STAT3 and STAT1	Gastric tumor gp130 Y757F/Y757F mice model	Upregulate the gene expression of MMP-13	(74)
IL-11	Under hypoxia conditions all cell lines upregulate gene expression and protein production of IL-11 Recombinant IL-11 treatment increased the migration and invasion under normoxia and neutralizing antibodies and siRNA suppressed the migration and invasion. Recombinant IL-11 increased STAT3 phosphorylation and expression of MMP-2, MMP-3 and MMP-9	Human Breast cancer cell line: MDA-MB-231; colorectal carcinoma cell line: HCT116; non-small lung carcinoma: H1299; malignant melanoma cell line: A375 and hepatocellular carcinoma cell line: HepG2	Upregulate the gene expression of MMP-2, MMP-3 and MMP-9	(75)
IL-12	IL-12 treatment inhibited lung tumor growth, resulting in the long-term survival of lung cancer-bearing mice. Further examination revealed that IL-12 rapidly activated NK cells to secrete IFN-γ, resulting in the inhibition of tumor angiogenesis and MMP-9 transcript level decreased MMP-9/TIM modulation in the tumor microenvironment after 14 days of IL-12 therapy produce reversible antitumoral effects	Murine breast cancer HTH-K (syngeneic breast carcinoma), injected in C57BL/6 mice to generate an orthotopic lung cancer model	L-12 prevented blood vessel regrowth and inhibit MMP-9	(76, 77)
IL-17	In breast tumors was observed the presence of IL-17 strongly positive cells within the scattered tumor-associated inflammatory infiltrate. IL-17 addition to breast cancer cell lines promoted significant invasiveness	Human Archival paraffin-embedded sections of 19 primary invasive breast tumors (15 Grade III and four Grade II). Human Breast cancer cell lines: MDA-MB231 and MDAMB435 cell lines	Selective antagonists for MMP-2/MMP-9 or MMP-3 suppressed the stimulatory effect of IL-17 on breast cancer invasion. However, IL-17 does not affect secretion of these MMPs	(78, 79)
IL-17	High salt synergizes with sub-effective IL-17 to induce breast cancer cell proliferation mediated activation of SIK3 (a G0/G1-phase inductor) by mTOR complex. SIK3 induce expression of CXCR4 through MMP-9 activation	Human Breast cancer cells lines: MCF7, MDA-MB-231, BT20, AU565	MMP-9	(80)
IL-17	MMP-7 mediates IL-17's function in promoting prostate carcinogenesis through induction of EMT, indicating IL-17-MMP-7-EMT axis as potential targets for developing new strategies in the prevention and treatment of prostate cancer	Murine Prostate cancer cell lines (LNCaP, C4-2B and PC-3). PB-Cre4 mice	MMP-7	(81)
IL-17B, IL-17RB	IL−17B dose dependently promoted the invasion, growth and migration of thyroid cancer cells. IL-17RB induced ERK1/2 activation pathway and increased MMP-9 expression Thyroid cancer cells express IL-17RA, IL-17RB and IL-17RC	16 paired Human thyroid cancer tissues Thyroid epithelial cell line HTori-3 and the thyroid cancer cell lines TPC-1, SW1736 and FTC-133	MMP-9	(82)
IL-17	IL-17A treatment promotes OE19 cell migration and invasion, upregulates MMP-2 and MMP-9 expression, increase ROS production, IκB-α phosphorylation and NF-κB nuclear translocation. IL-17 cause these effects through ROS/NF-κB/MMP-2/9 signaling pathway	Human Esophagus adenocarcinoma Cell line OE19	MMP-2 and MMP-9	(83)
IL-18, IL-10 and TNF- α	IL-18 and IL-10 synergistically act to amplify OPN and thrombin production, which in turn augments M2 macrophage polarization. M2 Macrophages and endothelial direct cell- cell interaction resulting in excessive angiogenesis	Mouse leukemic monocyte Mphi cell line RAW264.7 and Mouse endothelial cell line b.End5	Stimulation of RAW264.7 cells with TNF- α increases MMP-2 and MMP-9 gene expression IL-18 and/or IL-10 had no impact in the gene expression of MMP-2, MMP-3, MMP-7 and MMP-9	(84)
IL-32α	IL-32 stimulation in MG-63 cells shown, dose-dependently promoted the invasion and motility of osteosarcoma cells and induced the activation of AKT in a time-dependent manner. IL-32 stimulation increased the expression and secretion of MMP-13	Human MG-63 osteosarcoma cell line	MMP-13	(85)
IL-33	IL-33 increases the abilities of proliferation, migration and invasion of melanoma cells and Vasculogenic mimicry tube formation through ST2. IL-33 induces the production of MMP-2/9 via ERK1/2 phosphorylation	Human Melanoma of patients	MMP-2 and MMP-9	(86)
IL-33	IL-33 significantly promoted cell invasion and migration and induced the expression of MMP-2 and MMP-9 via ST2 and AKT pathway	Human Lung cancer cell lines: A549 and NCI-H1299	MMP-2 and MMP-9	(87)
IL-33, IL1RL1 (IL-1-R4)	IL-33 expression in the tumor epithelium of adenomas and carcinomas and expression of the IL-33 receptor, its receptor IL1RL1 in the stroma of adenoma and both the stroma and epithelium of human colorectal cancer IL-33 signaling promotes tumor growth and associated angiogenesis	Human colorectal cancer and mouse model of intestinal tumorigenesis	MMP-1 and MMP-3	(88, 89)
IL-35	IL-35 can induce N2 neutrophil polarization (protumor phenotype) by increasing G-CSF and IL-6 production, and promote Neutrophil infiltration into tumor microenvironment. IL-35 stimulated macrophages to secrete proinflammatory cytokines IL-1β, IL-6 and up-regulate the expression of MMP-9, suggesting antitumoral activity	Murine H22 hepatocarcinoma cell Line and B16F0 melanoma cells	MMP-9	(90)
IL-35	Significantly lower expression of IL-35 was also observed in Hepatocellular carcinoma patients. IL-35 over-expression in HepG2 cells significantly upregulated HLA-ABC and CD95, reduced activities of MMP-2 and MMP- 9, and decreased cell migration, invasion and colony formation capacities	Hepatocellular carcinoma from 75 patients and Human Hepatocellular carcinoma cell line HepG2	MMP-2 and MMP-9	(91)
IL-37	Transfected cells A549 overexpressing IL-37 cause low gene expression of MMT-9, PCNA, Ki-67, Cyclin D1 and CDK4, but elevated expression of caspace-3 and caspace-9. IL-37 inhibits the proliferation, migration and invasion of human lung adenocarcinoma A549 cells as well as the chemotaxis of Treg cells and promotes apoptosis of A549 cells	Murine Lung adenocarcino line cells A549. Xenograft mouse models.	MMP-9	(92)
CXCR4	Lymph node metastatic Hepatocarcinome Hca-F exosomes (contain elevated CXCR4) promote migration and invasion in HcaP cells elevating the secretion of MMP-9, MMP-2 and VEFG-C	Murine hepatocarcinoma cell lines Hca-F and Hca-P	MMP-2 and MMP-9	(93)
TNF- α	Tumor necrosis factor-α (TNF-α) induce a dose-dependent increase in MMP-9 activity HT1376 cells, through ERK1/2 and P38 MAP kinase activity and activation of the transcription factors NF-kB, AP-1 and SP-1	Human Bladder carcinoma cell line, HT1376	MMP-9 induced by TNF-α thought the NF-kB, AP-1 and Sp-1 cis-elements of the gene promoter mediated regulation ERK1/2 and p38 MAP kinase	(94)
TNF- α	TNF-α secretion from cancer cell line increased expression of MMP-2 and MMP-9 and increased TNF-α production. A TNF-α/TNF-R1/NF-kB system signaling pathway generated a highly metastatic cancer cells. TNF-α-triggered NF-κB activation to upregulation of active MMP released from the cancer cells	Human Oral squamous cell carcinoma SAS cell line. Metastatic cervical lymph nodes and metastatic lung cell lines induced by a SAS injected in the tongue of mouse	MMP-2 and MMP-9	(95)
TNF- α	Aberrant TNF-α signaling promotes cancer cell motility, invasiveness, and enhances cancer metastasis mediated NF-kB signaling. TNF-α-induced expression and stabilization of C/EBPb depends on p38MAPK activation, but not on NF-kB activity. C/EBPβ and its downstream MMP-1 and MMP-3 are required for TNF-α-induced cancer cell migration. TNF-α activates multiple signaling pathways, including NF-kB and C/EBPβ to promote cancer cell migration. TNF-α treatment significantly increased the number of migrated MDA-MB-231 and MDA-MB-435 cells in a dose-dependent manner	Human Breast cancer lines cells: MDA-MB-231 and MDA-MB-435	MMP-1 and MMP-3 mediate TNF-α-induced cell migration downstream of C/EBPb	(96)
TNF- α	AMB cells stimulation with TNF-α increased IL-6 and MMP-9 mRNA expressions, via NF-kB activation. Furthermore, TGF-β and IFN-c increased TNF-α-mediated expressions of MMP-9 and IL-6 mRNA, while those responses were suppressed by NF-kB inhibitor	Ameloblastoma cells (AMB) cultures from patients were inmortalized using hTERT vector	MMP-9	(97)

**Table 2:** Proteins associated with MMPs in angiogenesis on cancer.

**Protein**	**Intervention/process**	**Tumor type**	**MMP**	**References**
L1 adhesion molecule /CD171	Constitutive cleavage of L1 proceeds in exosomes mediated by a disintegrin and MMP10, under apoptotic conditions multiple MMP are involved	Human ovarian carcinoma cells OVMz	ADAM10	(98)
PIGF	Knockdown of PlGF in spheroid body cells reduced *in vitro* tumorigenicity and stemness properties (self-renewal ability, colony forming, migration and MMPs activities) and decreased ability to differentiation and angiogenesis	Human Spheroid cells from gastric adenocarcinoma MKN-45 and GS cells lines	MMP-2 and MMP-9 activities	(99)
VEGFR2 blockade	Brain tumor vessels: Vascular stabilization by increases pericyte coverage, up-regulation of angiopoitin-1 and collagenase IV activity provides and oxygenated environment through the degrades pathologically thick basement membrane by MMPs activation	Human Orthotopic glioblastoma obtained by xenografts on mouse of U87 gliomas tumors	MMPs−2 and MMP-9	(100)
VEGF	In colorectal liver metastasis, the high expression of stroma-derived MMP-12 and VEGF correlated with a dismal prognosis	Colorectal liver metastasis of patients	MMP-12	(101)
Angiopoietin-2	In colorectal lung metastases, the high stromal expression: MMP-1-2,-3 is indicator for a more favorable clinical outcome, whereas high expression of stromal angiopoietin-2 is associated with a reduced cancer-specific survival and an independent prognostic marker for cancer-specific survival in lung metastasis	Colorectal lung metastasis of patients	MMP-1, MMP-2 and MMP-3	(101)
Chemokines related to the immune system and ENPP3, BNIP3, AZGP1 and PIGR	Stage II colorectal cancer. Poor prognosis is associated with low expression of the genes PIGR, CXCL13, MMP3, TUBA1B, CXCL10, and high expression of SESN1, AZGP1, KLK6, EPHA7, SEMA3A, DSC3 ENPP3, BNIP3 and ENPP3	Human Stage II colorectal cancer	MMP3	(102)
MIF	Increased expressions of both MIF and MMP-9 were significantly associated with microvessel density of tumor, but only dual high-expression of MIF and MMP-9 was in relation to tumor invasion and tumor recurrence	67 intracranial meningioma from patients; 57 benign tumors (WHO grade I) and 10 non-benign (WHO grade II/III)	MMP-9	(103)
TGF-β	TGF-β-pretreated A549 cells increased migration and invasiveness, decreased expression of E-cadherin, tight-junction proteins and increased expression of N-cadherin and vimentin. TGF-β-mediated exosomes and might function by increasing the expression of MMP-2	Human Carcinoma lung A549 cell line	MMP-2	(104)
TGF-β, IL-1α	Production of IL-1a by pancreatic stellate cells induce alterations in MMP and TIMP profiles and activities, upregulating MMP-1 and MMP-3. TGF-β counteracted the effects of IL-1α on pancreatic stellate cells downregulating and reestablishing MMP and TIMP profiles	Pancreatic Stellate Cells from patients with pancreatic ductal adenocarcinoma	MMP-1 and MMP-3	(105)
EGR1	EGR1 mediates hypoxia-induced SIRT1 transcriptional repression, and the acetylation of NF-kB and the activation of MMP-2 and MMP-9	Human HCT 116 and SW480 Cell colorectal cancer cells line	MMP-2 and MMP-9	(106)
CHI3L1 (Chitinase 3-like protein 1)	CHI3L1 promotes the metastasis of gastric and breast cancer cells, interacts with the IL-13Rα receptor on the plasma membrane of gastric cancer cells. Even more, CHI3L1 activates MAPK signaling pathway in gastric and breast cancers and the activator protein-1 (AP) transcriptional activity in cancer cells	Gastric cancer cells: MKN-45, AGS, MGC-803 and HGC-27. Breast cancer cells: MDA-MB-231, MDA-MB-435 and MDA-MB-468. Melanoma cells (A375)	MMP-1, MMP-2, MMP-3, MMP-7, MMP-9, MMP-12	(107)

Angiogenesis studies using MMP-8 and MMP-2 knock-out mice, show an *in vitro* reduction of cell proliferation and neocapillary network growth, as well as a decrease in HUVEC migration and poor *in vivo* angiogenesis. Interestingly, ischemia-induced neovascularization is also affected by a reduction in ECs and invasive, proliferative, or mobilizing activities of endothelial progenitor cells (EPCs) derived from bone marrow (113, 114). Additionally, it is known that ECs secrete MMP-2 and−9-containing vesicles stimulated by VEGF and FGF-2 and thus regulate the proteolytic activity critical for the angiogenesis-related invasive and morphogenic processes (115). Furthermore, MMP-9 also generates the angiogenic and tumoral repressor, tumstatin by proteolysis of the non collagenous domain (NC1) from the collagen alpha-3(IV) chain. The anti-angiogenic properties of tumstatin inhibit EC proliferation and induce apoptosis by interacting with alphaVbeta3 integrin (116).

**Figure 3:**
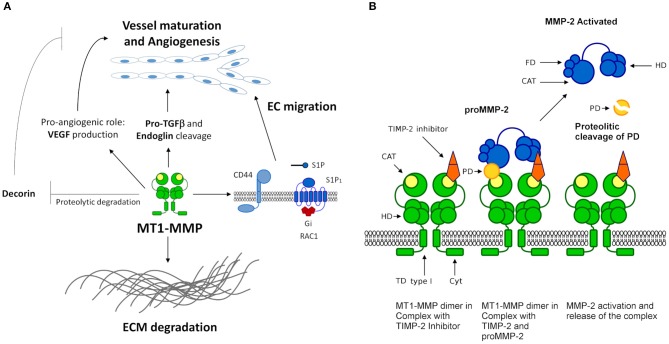
MT1-MMP functions and mechanism. **(A)** MT1-MMP (MMP-14) participates in angiogenesis regulation and remodeling of the ECM. MT1-MMT interacts with cell surface molecules such as CD44, S1P_1_ (G-protein coupled receptor coupled to the G(i) subclass of heteromeric G proteins) and receptors such as discoidin domain receptor (DDR1). S1P represents sphingosine-1-phosphate ligand of S1P_1_ leading to the activation of RAC1. MT1-MMT cleaves collagen type I to prevent DDR1 recognition and the apoptotic cascade. MT1-MMT is a key effector in the production of pro-angiogenic factors such as VEGF and is able to degrade pro-TGF-β and endoglin (TGF-β receptor), suggesting a pivotal role in vessel maturation and angiogenesis, respectively. **(B)** MT1-MMP model of the interaction and activation of pro-MMP-2. MT1-MMP forms a homo-dimer in the membrane mediating the interaction of the hemopexin and the transmembrane domains, necessary conditions for the activation of pro-MMP-2. MT1-MMP dimer forms a complex with one TIMP-2 inhibitor, the interaction is not a symmetric array. TIMP-2 binds to a single MT1-MMP monomer by the catalytic domain mediated by the N-terminal. The C-terminal of TIMP-2 binds to the hemopexin domain of pro-MMP-2, thus allowing the prodomain of MMP-2 to access the catalytic domain of the second monomer of MT1-MMP.

Among the most studied MMPs participating in angiogenesis is the MMP-14 (MT1-MMP). It significantly contributes to angiogenesis regulation by cleaving ECM molecules as a matrix-degrading enzyme (Figure 3). This MMP also acts as a key effector in the production of pro-angiogenic factors such as VEGF. In addition, MT1-MMP interacts with cell surface molecules, such as CD44 and sphingosine 1-phosphate receptor 1 (S1P_1_), to induce EC migration, and plays a critical role in the proteolytic degradation of anti-angiogenic factors as decorin. Furthermore, evidence shows that MT1-MMP is able to degrade pro-TGF-β and endoglin (TGF-β receptor), suggesting a pivotal role in vessel maturation and angiogenesis, respectively (117) (Figure 3A). In addition, MT1-MMP appears to be an essential molecule that determines ECM adhesion and human endothelial cell tube formation through the modulation of MMP-2 expression (Figure 3B). This suggests an important role in regulating angiogenesis-related functions in human ECs (118).

### Soluble MMPs in Cancer Angiogenesis

Soluble MMP expression and its effects on cancer stabilization/proliferation are intimately linked via vascular angiogenesis mechanisms that are now well recognized. In this regard, MMP-1 expression has been reported to contribute to the progression of Head and neck squamous cell carcinomas (HNSCC) and the suggest metastatic phenotype of human breast and colorectal cancers, among others (119–121). Interestingly, MMP-1/protease-activated receptor-1 (PAR1) signaling axis has been implicated in tumor angiogenesis and intravasation of carcinoma cells by inducing vascular permeability (122), as well as, hypoxia-regulated MMP-1 expression in metastatic bladder cancer cells, which could be associated to a reactive oxygen species (ROS)-related regulation of the spheroid metastatic phenotype and cell spread (123). The increased expression of MMP-1 in human chondrosarcoma is an important prognostic factor and its function in the spread of tumor cells has been evaluated by silencing assays in which cancer metastasis is impaired but local tumor growth and angiogenesis are enhanced (124). These findings strongly support a role for MMP-1 in the diverse proliferative outcomes of human cancer through angiogenic processes.

Many studies have been published describing the relationship between MMP-2 expression and tumor angiogenesis. One of the earliest reports indicates that IL-8, an angiogenic factor, induces MMP-2 expression and activity in melanoma cells, enhancing their invasion (125). A relationship between MMP-2 expression and stromal support, angiogenesis, invasiveness, and tumor growth was demonstrated using an MMP-2-specific inhibitor in a mouse model of bladder cancer (126). Furthermore, an elevated expression of MMP-2 was correlated with VEGF expression in gastric cancer (127) which suggests that this MMP plays a critical role in the progression of cancer through ECM degradation, tumor neovascularization and metastasis.

On the other hand, MMP-9 promotes endothelial cell migration and triggers the angiogenic switch by releasing VEGF during carcinogenesis (128). Decreased expression of VEGF and MMP-9 in medulloblastoma cells that overexpress osteonectin, also referred to as Secreted Protein Acidic and Rich in Cysteine (SPARC), leads to decreased angiogenesis and tumor growth, indicating the pro-angiogenic role of MMP-9 in cancer tissues (129). In contrast, the direct proteolytic cleavage of osteopontin (OPN) by MMP-9 contributes to cancer metastasis, most likely associated with angiogenesis via the regulation of VEGF and angiostatin secretion (130, 131). This model suggests that cancer growth is accompanied by increased vascular permeability, due in part to the expression of MMP-9, leading to the regulation of angiogenic factors, and eventually, neovascularization in cancer tissue.

Studies have revealed that, both MMP-2 and MMP-9 can degrade type IV collagen and are frequently elevated in human cancer. Additionally, a cooperative effect of MMP-2 and MMP-9 was demonstrated in an *in vivo* experimental model establishing the angiogenic phenotype and invasiveness of tumor keratinocytes (132). The mechanism whereby MMP-2 and MMP-9 activity induces cancer angiogenesis involves the cleavage of latent TGF-β in a CD44-dependent manner, which can promote tumor growth and invasion (133). Together, these results confirm the contribution of MMP-2 and MMP-9 to cancer angiogenesis through the degradation of ECM components and the activation of pro-angiogenic factors VEGF and TGF-β in diverse cancer tissues (134). The above findings may explain the central role of the metalloproteinases MMP-2 and MMP-9 in tumor angiogenesis through the induction of pro-angiogenic factors.

Many other MMPs have also been implicated in the incipient establishment of cancer angiogenesis (Tables 1, 2). For example, MMP-3 and MMP-7 interact *in vivo* with osteopontin at tumor sites and may be related to the angiogenic process during tumor development (135). The interaction of diverse MMPs with another class of proteases also contributes to tumor angiogenesis. For example, the overexpression of the serine protease matriptase in human carcinoma cells regulates MMP-3 activity, promoting proliferation and angiogenesis of tumor tissues by degradation of surrounding ECM (136). MMP-13 has also been implicated in cancer angiogenesis promotion through tube formation and neo-capillary network development mediated by stimulation of ERK-FAK signaling pathway stimulation. It also stimulates VEGF-A secretion, which contributes to the angiogenic process (137).

It has been widely accepted that MMPs likely play antagonistic roles in regulating cancer angiogenesis. MMP-7 and MMP-9 may be involved in the blockage of cancer angiogenesis by cleaving plasminogen and generating angiostatin molecules (138). Additionally, cross-talk between MMP-7 and MMP-9 leads to the cleavage of insulin-like growth factor-binding protein 2 (IGFBP-2), an angiogenic activator in major aggressive cancers via the transcriptional regulation of the VEGF gene, showing adverse effects in cancer angiogenesis in some tissues (139, 140). It has also been described that MMP-19 is essential to the development of nasopharyngeal carcinoma due to its tumor suppressive and anti-angiogenic functions which can reduce secreted MMP-2 and VEGF (141).

### Membrane-Type Metalloproteinases (MT-MMPs) in Cancer Angiogenesis

Membrane type 1 matrix metalloproteinase (MT1-MMP) is considered a key mediator of cancer progression and metastasis. The overexpression of MT1-MMP in malignant breast cells significantly enhances VEGF production via the Akt and mTOR signaling pathways activated by the MT1-MMP–VEGFR-2–Src complex, which promotes tumor growth and angiogenesis (142, 143). Apparently, a similar mechanism could be involved in glioblastoma angiogenesis (144, 145). Therefore, it is worth noting that this tumor phenotype appears to be associated with the dependence of Akt-mediated signaling pathway, which is stimulated by several angiogenic factors.

On the other hand, the proteolytic cleavage of semaphorin 4D into its soluble form by MT1-MMP provides a novel molecular mechanism to control tumor-induced angiogenesis in HNSCC (146). In addition, cross-talk between MT1-MMP, MMP-2, and laminin-5γ2 chain fragments contributes to the vasculogenic mimicry of melanoma cells (147). In contrast, the colorectal cancer cells that shed MT1-MMP–mediated endoglin fragments exhibit an anti-angiogenic effect (148). Other studies have demonstrated that MT1-MMP and membrane type 2 matrix metalloproteinase (MT2-MMP) work cooperatively as pro-invasive factors that directly lead to Snail1-triggered cell participation in cancer angiogenesis and metastasis (149). In addition, MT2-MMP is a potential EMT mediator in carcinomas that can degrade adherents and tight junction proteins (150). It has been reported that both MT1-MMP and membrane type 3 matrix metalloproteinase (MT3-MMP) modulate pro-MMP-2 activation, whose angiogenic role in cancer was mentioned above, through inhibition by TIMP-2 and TIMP-3 (151). These data show that a cooperative effect of MT-MMPs during cancer angiogenesis is required together with the angiogenic factors.

Moreover, MT4-MMP expression correlates with EGFR activation, which triggers an angiogenic switch through its catalytic activity and induces the dissemination of cancer cells by disturbing the vessel integrity of the primary breast tumor and promoting hematogenous but not lymphatic metastasis (152–154). Finally, it has been shown that a high MT6-MMP expression in cancer cells is associated with tumor growth; however, further experiments are necessary to determine the exact role of this MT-MMP in the angiogenic process (155).

### MMPs and the Immune System in Cancer

It is known that uncontrolled angiogenesis, anomalous ECM turnover, decreased growth, and cell migration, as well as inflammatory response, are the result of an imbalance between MMPs activity and their inhibitors, which may be associated with different diseases. Several specific signals are responsible for coordinating the formation, growth, remodeling, and stabilization of blood vessels. It is recognized that excessive growth-promoting signal cues lead to pathological angiogenesis and cancer (15, 156).

In the tumoral microenvironment, there is a complex and dynamically interacting areas involving stromal cells (fibroblasts, myofibroblasts, neuroendocrine cells and immune cells), blood vessels, lymphatic network, and ECM (157, 158), resulting in a tremendous heterogeneity observed in cancer cells. This condition is because tumor cells express and modulate a broad group of signaling pathways including immune modulatory pathways of cytokines and chemokines, which participate in the progression and establishment of cancer cells (Tables 1, 2) (159–169). In this regard, both cytokines and chemokines induce the expression and activity of MMPs, which in turn allow for the activation of pro-inflammatory signaling pathways as well as the activating receptors, for example, expressed on the surface of T cells and NK cells. Of these molecules act as a powerful mechanism to regulate the immune response. We have analyzed and highlighted several studies showing the involvement of MMPs and their interactions with immune system proteins in angiogenesis and cancer processes as shown in Tables 1, 2.

The presence of secreted extra-cellular vesicles (exosomes) has recently gained importance within the tumor microenvironment (170–172). Exosomes are specific bearers of multiple modulating molecules, such as the antigens for cluster of differentiation (CD), cytokines and chemokines, growth factors (EGF, FGF, PIGS), adhesion proteins (L1/CD171), nucleotides (non-coding RNA, miRNA), and metalloproteinases. Exosomes activate and modify the activity of diverse proteins such as immune proteins and receptor ligands into the circulation by proteolytic cleavage, playing a role as effectors and regulators to promote crosstalk between cancer and stromal cells (53, 157, 173–176). In addition, *in vitro* studies revealed that a type of TGF-β mediated exosome derived from lung cancer cells increase the expression of MMP-2 (104), while immunosuppressive exosome secretion from lymphoblastoid cells induces apoptosis in CD4^+^ T cells (177). Therefore, exosomes could function as conversion markers of malignant cells.

Since they are diverse, MMPs influence multiple cellular processes, such as the inflammatory process regulating barrier function and the activity of inflammatory cytokines and chemokines. Chemoattractant proteins such as MCP-1, MCP-2, MCP-3, and MCP-4 are targets for MMP activity, as result the modified MCPs changing their activity from agonist to antagonist and causing inflammation. Inflammation produces immune tolerance and leads to specific micro-environment conditions, exploited by tumors to evade immune cells and enhance progression, angiogenesis and metastasis (78, 171). In the inflammation process, FGF2 expression can facilitate induction of FGF-dependent angiogenesis by mononuclear phagocytes, T-lymphocytes, and mast cells. The mechanism mediated through FGF2 release induces pro-inflammatory molecules, such as IFN-α, IL-2, IL1-β, and nitric oxide. During the first phases of the EC angiogenesis process, FGFs (1, 2, and 4) upregulate urokinase-type plasminogen activator (uPA) *in vitro* and transform plasmin into plasminogen, an activator of MMPs, triggering ECM degradation and the secretion of exosomes containing MMP-2, MMP-9, TIMP-1, and TIMP-2 (156, 178).

Several studies, both clinical and experimental, have shown that elevated MMP (including MT1-MMPs) levels are associated with the modulation of tumor progression. In brain tumors, growth factors and cytokines modulate the activity of several MMPs. Additionally, it has been observed that MMP-2-positive tumor cells in patients are correlated with low mean survival (54, 179, 180).

Furthermore, in the context of immune cells, there are tumor-associated cells that contribute to the synthesis and upregulation of MMPs (70, 181–183). Importantly, tumor-associated macrophages (TAMs) secrete membrane-bound or soluble proteases, such as MMP-2, MMP-9, and MMP-12, which are involved in ECM degradation and promote the infiltration of tumor-associated blood vessels (184). Macrophages are known to promote cancer initiation and tumor development in an inflammatory environment (185). Bone marrow-derived myeloid cells are also involved in the process, through active regulation of blood vessel formation and maintenance in tumors (186).

Furthermore, accumulated evidence shows that primary tumors can recruit immune cells, such as MMP-9 positive neutrophils, B cells, and M2 polarized macrophages to produce tumor-associated immune cells, which are known to contribute to neovascularization by supplying MMP-9 and other MMPs (59–61, 90, 183, 187). Although most of the published studies consider that M2 macrophages produce high amounts of IL-10, IL-1β, VEGF and MMP, additional subsets have been described with different proportions of cytokines related to cancer microenvironment. A subset of high M1 and low M2 infiltration macrophages are associated with improved patient survival in non-small-cell lung cancer (188), while the activation of M2 macrophages is correlated with a negative prognosis in cancer progression (189). It is therefore important to reorient the associated functions of the M2 macrophage subset to stop and kill cancer cells.

Finally, the molecular role of MMPs in the immune system and cancer is to modulate a series of latent signaling proteins located in ECM, including cytokines and growth factors such as quiescent TGF-β forming a complex with TGF-β-binding protein-1 in ECM. Thus, TGF-β modulates MMP expression, resulting in a bidirectional regulatory loop enhancing TGF-β signaling and promoting cancer progression (133, 190–194). Another mechanism observed is MMP-9 activity, which truncates IL-8 (1–19, 21, 22, 26–52, 63, 108–135) into more active chains, altering the function of the receptor and improving its biological activity, resulting in greater chemotaxis for neutrophils than the intact form of theof cytokine (195, 196).

Accordingly, immunomodulatory mechanisms of MMPs, cytokines, receptors, and growth drivers are involved in the development and progression of several types of cancer.

## Therapeutic Perspective of MMPs

MMPs and their inhibitors TIMP, control a wide variety of physiological processes. They constitute promising pharmaceutical targets for inhibition and other metastatic processes.

Currently, monoclonal antibodies are possible candidates to inhibit the activity of MMPs (MMP−14,−12,−9, and−2). However, studies have only managed to identify antibodies against MMP-9 activity, which has biological functions and not for the MMP−14,−12, and−2 (197). In prostate cancer, MMP-9 may amplify local angiogenesis by cleaving membrane-bound VEGF. Therefore, VEGF is a candidate to be blocked and controlled and to prevent the activation of the androgen receptor (AR)/phosphatidylinositol 4-phosphate 5-kinase type-1 alpha (PIP5K1α)/AKT/MMP-9/VEGF signaling axis required for cell survival and invasion of metastatic tumors (198). Similarly, the effect of phytochemicals on MMP-2, MMP-9, and their tissue inhibitors (TIMPs) has been tested in breast cancer, with no alterations observed *in vitro* (199). TIMP-3 has been another important target of study regarding the inhibition of cancer cell migration, invasion, and metastasis *in vitro* and *in vivo* by natural products (200).

Recently, the effect of MMP-2 gene silencing in normal and MCF-7 cells exposed to the irradiation has been studied. It is known that this MMP leads to the degradation of basement membranes; however, the differential response to DNA damage silencing the MMP-2 gene in normal and MCF-7 cells may be attributable to ROS generation (201). In addition, thrombospondin-2 (THBS2) is a target gene of microRNA-93-5p (miR-93-5p) and THBS2 is closely associated with ECM and MMP-2 and 9. This MicroRNA is involved in the progression of malignant tumors and is highly expressed in cervical cancer tissues and cells. Thus, the THBS2/MMP signaling pathway is relevant for more studies in clinical trials (202). Moreover, the combined therapy for glioma treatment using temozolomide-marimast (a specific alkylating agent and an MMP inhibitor, respectively) results in tumor cell progression and invasiveness. An alternative treatment proposed in an *in vitro* study uses a combination of temozolomide and compounds 1 and 2 of N-O-isopropyl sulfonamido-based hydroxamates (MMP-2 inhibitors) to inhibit cancer cell invasiveness and viability (203).

All these studies represent advances in cancer drug development and cancer therapy, with a focus on the control of MMPs and the proteins with which they form complex networks of multifunctional interactions to modulate the signaling pathways that deviate during the development of metastatic cancer. Importantly, the emerging combined clinical therapy mitigates the side effects of existing treatments and raises the anticancer efficacy of chemotherapeutic drugs.

## Conclusion Remarks

In this work, we have highlighted the role that MMPs play in the cancer and its interaction with growth factors, inhibitor proteins, and the EMT process. The activity of MMPs is involved in the degradation, remodeling, and exchange of ECM, which, under normal physiological conditions, contributes to homeostasis as part of an extensive network of extracellular tissue modulation. In cancer, homeostasis is modified, leading to localized abnormal physiological conditions that modify this extensive network of extracellular tissue modulation.

The increase in MMP activities, as an abnormal process, is a way of producing/inducing an erroneous metabolic cascade. Erroneous metabolic cascades are signals that trigger the emergence of complex abnormal cell pathways, which give rise to tumor/cancerous phenotype cells. In this regard, the transformation into tumor/cancerous phenotypes suggests an exacerbated adaptive survival process. MMPs are not the only elements of this extensive network of extracellular tissue modulation; others such as TIMP proteins, which modulate MMPs, ADAMs and ADAMTSs (15), play a role in this anomalous process as numerous regulatory branches of the network do, namely interleukins.

Although the information on the role of MMPs in cancer is very broad and the way these proteins are expressed is well known, we observed a significant lack of data at the fine level of the relationship between MMPs and the continuity of both the normal and altered signals that positively modulate the carcinogenic process. MMPs produce modulatory elements that remain unclear and, considering that the ECM is a complex array of proteins, fibers, and carbohydrates in different tissues, there may be several variants that generate the loss of homeostasis, causing the diverse cancerous processes observed.

In the angiogenesis process, MMPs are well-known key factors involved in ECM degradation that induce angiogenesis initiation in both physiological and pathological processes. However, the experimental evidence thus far demonstrates that MMPs also play a decisive role in the activation of pro-angiogenic and, in some cases, anti-angiogenic factors in cancer tissues. Thus, MMPs can be considered angiomodulators, which could control new vessel formation necessary for cancer growth, progression, and spread. Therefore, we speculate that MMPs participate in cancer angiogenesis in a cell context-dependent manner.

Most of the experimental data regarding MMP participation in cancer development, vascular endothelium processes, other epithelia (such as periodontal), and inflammatory processes allow us to assume that MMPs are proteins that carry out a type of external cellular regulation/signaling on the ECM. These proteins possess a different regulatory action mechanism that is complementary to other mechanisms such as ligand-receptor signaling pathways. The MMP mechanism is based on editing macromolecules by proteolysis, mainly anchored to the ECM.

It is evident that the proportion of MMPs and other macromolecules (cytokines, grown factors, fibers such as integrins, polysaccharides, and others) in the ECMs of different tissues in normal conditions are metabolically, micro-environmentally, and epigenetically balanced for their functions in each type of tissue. There are tissue-specific proportions, although ECMs have a high degree of heterogeneity.

Inspecting our concept of MMP participation in ECM in the literature, we found an excellent review of ECM with similar concepts (204). The ECM is a highly dynamic system in constant remodeling and is undoubtedly an extension of communication/modulation/signaling among cells located outside the plasma membrane, where MMPs participate in protein editing by providing post-translational modifications.

On the other hand, EMT is a biological process aimed producing mesenchymal phenotype cells from epithelial cells. Its inverse process, mesenchymal-epithelial transition (MET), is carried out with the participation of ECM elements. EMT and MET lead to normal tissue regeneration and fibrosis [EMT type 2, according to Kalluri (205)]. Regarding the participation of MMPs in EMT type 3 (abnormal type), it is evident that their participation is associated with errors in the communication, modulation, and signaling of this process, which is induced and directed by tumor cells.

Evidence suggests that tumor cells induce the uncontrolled upregulation of MMPs, producing a large number of stimulating factors that disrupt EMT and immunological processes that prevent tumor cell elimination and migration. The upregulation progresses to generating anomalous tissue-specific type signals.

It is known that tumor cells have extensive heterogeneity in their metabolism and phenotype relative to normal tissue across cancer types. Furthermore, these abnormal signals coming from the tumor cells are tissue-specific, leading to the adaptation to the microenvironment where they developed. Finally, an interesting observation of the MMP family is the large, robust specificity profile, which suggests that its role is controlled in a tissue-specific manner; that is, MMP types are expressed accordingly to the regulatory proteins needed for the tissue.

However, more research efforts are needed to determine when abnormal signals begin, what the determinants are, and how microenvironmental tissue-specific conditions can lead a cell to change its metabolism and phenotype. In addition, the question remains: When does the high expression problem of MMPs become a problem metabolically? Although MMPs do not seem to be the cause of the appearance of tumor cells, they induce tumor development because they are targets to regulate development, contributing to increased invasiveness and growth of metastatic tumors.

## Author Contributions

MA-S conceived the idea and scripted the basis of the manuscript. RA and MA-S had equal contributions and a role in the design, analysis, and writing of the article. RA contributed in entirety to the design of the Figure 1. SQ-F conceived the idea. SQ-F and RA designed of the Figure 2, Table 2, and drafted the manuscript. RA conceived and designed the Figure 3B and participated with JT-R, VA-A, JL-R, MR-C, and MA-S in the designed the Figure 3A. EB-V participated in its construction. All authors reviewed the literature, critically reviewed the manuscript, and approved the final version.

### Conflict of Interest

The authors declare that the research was conducted in the absence of any commercial or financial relationships that could be construed as a potential conflict of interest.
